# Comparative Study on Pulmonary Toxicity in Mice Induced by Exposure to Unflavoured and Apple- and Strawberry-Flavoured Tobacco Waterpipe Smoke

**DOI:** 10.1155/2020/6450450

**Published:** 2020-01-14

**Authors:** Abderrahim Nemmar, Suhail Al-Salam, Sumaya Beegam, Priya Yuvaraju, Badreldin H. Ali

**Affiliations:** ^1^Department of Physiology, College of Medicine and Health Sciences, United Arab Emirates University, P.O. Box 17666, Al Ain, UAE; ^2^Zayed Center for Health Sciences, United Arab Emirates University, P.O. Box 17666, Al Ain, UAE; ^3^Department of Pathology, College of Medicine and Health Sciences, United Arab Emirates University, P.O. Box 17666, Al Ain, UAE; ^4^Department of Pharmacology and Clinical Pharmacy, College of Medicine and Health Sciences, Sultan Qaboos University, P.O. Box 35, Muscat 123, Al-Khoud, Oman

## Abstract

The use of flavoured tobacco products in waterpipe smoking (WPS) has increased its attractiveness and consumption. Nonetheless, the influence of flavourings on pulmonary toxicity caused by WPS remains unclear. Here, the pulmonary toxicity induced by plain (P)-WPS, apple-flavoured (AF)-WPS, and strawberry-flavoured (SF)-WPS (30 minutes/day, 5 days/week for 1 month) was investigated in mice. Control mice were exposed to air. Exposure to P-WPS or AF-WPS or SF-WPS induced a dose-dependent increase of airway hyperreactivity to methacholine. The histological evaluation of the lungs in all the WPS groups revealed the presence focal areas of dilated alveolar spaces and foci of widening of interalveolar spaces with inflammatory cells. In the lung, the activity of neutrophil elastase and myeloperoxidase and the concentrations of tumor necrosis factor-*α* and glutathione were increased by the exposure to P-WPS, AF-WPS, or SF-WPS. However, the levels of interleukin-6 and catalase were only increased in the AF-WPS and SF-WPS groups, while nitric oxide activity was only increased in the SF-WPS group. DNA injury was increased in all the WPS groups, but the concentration of cleaved caspase-3 was only elevated in the SF-WPS group. The exposure to either P-WPS or AF-WPS or SF-WPS increased the expression of nuclear factor kappa-B (NF-*κ*B) in the lung. In conclusion, the exposure to P-WPS or AF-WPS or SF-WPS induces alterations in lung function and morphology and causes oxidative stress and inflammation via mechanisms that include activation of NF-*κ*B. Overall, the toxicity of flavoured tobacco WPS, in particular SF-WPS, was found to be greater than that of unflavoured WPS.

## 1. Introduction

Waterpipe smoking (WPS), also commonly referred to as “shisha” or “hookah” that uses sweetened, flavoured tobacco products, has turned out to be a prevalent phenomenon not only in the Middle Eastern and Asian countries but also in European and North American countries [1]. In Eastern Mediterranean countries, the use of WPS among the youth has been reported to be between 2.5% and 37.2%; in European countries between 2.2% and 22.7%; and in the United States of America between 1.0% and 11.4% [1].

WPS is commonly smoked using a mixture of tobacco, glycerol, and supplementary additives and is commercialized with several flavours including fruits, candy, and beverages [1]. Moreover, charcoal is used to heat WPS tobacco in the waterpipe. The attractiveness of flavoured tobacco has been reported to be the main reason why youth begin smoking waterpipe and the misperception that flavoured tobacco products are safer than the unflavoured ones [2]. The latter phenomenon is not only seen with WPS but also extends to the usage of e-cigarettes among young people. The attractiveness of e-cigarettes has been related to the flavourings used in e-liquids of e-cigarettes [3]. The consumption of flavoured tobacco by waterpipe consumers is very frequent. In the USA, it has been reported that 82.3% of adult who smoked waterpipe in the past 30 days stated using a flavoured tobacco [4]. The same study has shown that the flavouring use was analogous between women (83.6%) and men (81.3%) [4]. However, despite their widespread use, little is known on the adverse health effects of the flavourings used in WPS.

In a recent systematic review, it has been reported that WPS was significantly associated with respiratory diseases, including chronic obstructive respiratory disease (odds ratio (OR) = 3.18, 95%confidence interval (CI) = 1.25, 8.08), bronchitis (OR = 2.37, 95%CI = 1.49, 3.77), and passive WPS and wheeze (OR = 1.97, 95%CI = 1.28, 3.04) [5]. Moreover, clinical and experimental studies have shown that WPS inhalation increases airway reactivity, decreases lung function, and induces inflammation and oxidative stress [6–8]. Nevertheless, the possible impact of flavourings on the respiratory pathophysiological effects that induced WPS remains largely uninvestigated by both clinical and experimental studies. Hence, the aim of the present study is to compare the pulmonary effects and mechanisms of action of unflavoured or plain (P), apple-flavoured (AF), and strawberry-flavoured (SF) tobacco WPS in exposed mice by assessing lung function, histopathology, oxidative stress, inflammation, DNA damage, and apoptosis.

## 2. Material and Methods

### 2.1. Animal Experimentation Protocols

All animal experimentation protocols were approved by the Institutional Animal Care and Use Committee of the United Arab Emirates University (Approval # ERA_20175625).

### 2.2. WPS Inhalation

Both male and female C57BL/6J mice (aged 6–10 weeks, weighing 20–25 g) (The Jackson Laboratory, Bar Harbor, USA) bred in the local central animal facility of the College of Medicine and Health Sciences, United Arab Emirates University were used. The animals were maintained in a temperature-controlled facility, with a 12 h light/dark cycle, and were provided access to water and food *ad libitum*. A total number of 101 mice was used in the present study, and the number of male and female mice utilized for the evaluation of the various parameters was comparably distributed among the studied groups.

After one week of familiarization to their conditions, the mice were indiscriminately separated into 4 groups, air (control), P-WPS, AF-WPS, and SF-WPS.

The WPS inhalation protocol has been performed according to recently described methods [9–11]. Mice were exposed using a nose-only exposure system (InExpose System, Scireq, Canada) to mainstream WPS generated by commercially available P- or AF- or SF-tobacco (Al Fakher Tobacco Trading, Ajman, UAE). For each daily session, 10 grams of tobacco was placed into the WPS head. At the end of WPS exposure session, the remaining tobacco was discarded. Control mice were exposed to air only. The duration of the session was 30 min/day and 5 days/wk for 1 mo [9–11]. The inhalation procedure was monitored by a computerized system. A computer-monitored puff was produced every 1 min (consisting of a 2 s puff time of WPS after that a 58 s of fresh air).

### 2.3. Airway Hyperresponsiveness to Methacholine

A sample size of 25 mice was used in this experiment. Airway hyperreactivity responses were evaluated in the air, P-WPS, AF-WPS, and SF-WPS groups using a forced oscillation technique (flexiVent, SCIREQ, Montreal, Canada). Airway resistance (*R*) was assessed after increasing exposures to methacholine aerosol produced with an inline nebulizer and given directly via the ventilator (0-40 g/ml), according to recently described techniques [10, 11].

### 2.4. Histopathology

For histological analysis, a sample size of 24 mice was used. After fixation of lung tissue in neutral buffered formalin (10% *w*/*v*) for a day, the tissue was gradually dehydrated in increasing concentrations of ethanol, cleared of alcohol residue in xylene, and lastly embedded in paraffin. Sections of 5 *μ*m (7 sections per lung) were cut with a microtome (RM2125 RTS, Leica Biosystems, Nussloch, Germany) and stained with hematoxylin and eosin [11, 12]. The stained sections were mounted on slides and evaluated blindly under a light microscope with a 40× objective lens by the histopathologist (SA) who contributed in this study. The morphometric analysis of neutrophil polymorphs and macrophages in lung tissues was carried out using ImageJ software (http://rsbweb.nih.gov/ij/). The level of interstitial infiltration by inflammatory cells was determined by counting the number of macrophages and neutrophils in 10 randomly selected high-power fields (HPF) in the lung sections. The mean numbers of macrophages and neutrophils were then converted from per HPF to per mm^2^ (each mm^2^ = 4 HPF). The calculation was carried out on random 10 HPF in each section in all the groups [13].

### 2.5. Estimation of Markers of Inflammation, Oxidative Stress, Cleaved Caspase, and Nuclear Factor Kappa-B (NF-*κ*B) in Lung Tissue Homogenates

For the biochemical analysis, a sample size of 32 mice was used. Following the exposure to either air or P-WPS or AF-WPS or SF-WPS, mice were sacrificed by an overdose of sodium pentobarbital, and their lungs were rapidly removed and rinsed with ice-cold PBS (pH 7.4) before homogenization, as reported earlier [7, 14]. The homogenates were centrifuged for 10 min at 3000 × *g* to eliminate cellular debris, and the supernatants were utilized for supplementary analysis [7, 14]. Protein content was quantified by Bradford's method.

The activities of neutrophil elastase (Cayman Chemical, Michigan, USA) and myeloperoxidase (MPO) (R&D Systems, Minneapolis, MN, USA) were measured using commercially available kits. The determination of total nitric oxide (NO) was performed with a total NO assay kit from R&D Systems (Minneapolis, MN, USA), which measures the more stable NO metabolites NO_2_^−^ and NO_3_^−^. The activity of catalase (Cayman Chemical, Michigan, USA) and reduced glutathione (GSH) concentration (Sigma-Aldrich Fine Chemicals) were measured according to the vendors' protocols. The concentrations of interleukin-6 (IL-6) and tumor necrosis factor-*α* (TNF*α*) were determined using commercially available kits (DuoSet, R&D Systems, Minneapolis, MN, USA).

The quantification of cleaved caspase 3 in lung tissue homogenates obtained from mice exposed to either air or P-WPS or AF-WPS or SF-WPS was performed using enzyme-linked immunosorbent assay using the commercially available kit obtained from R&D Systems (DuoSet, Minneapolis, MN, USA).

The estimation of NF-*κ*B levels in lung tissue homogenates obtained from mice exposed to either air or P-WPS or AF-WPS or SF-WPS was performed using the commercially available enzyme-linked immunosorbent assay kit obtained from Cell Signaling Technology (Danvers, MA, USA). The obtained measurements were expressed as optical density using a microplate reader with a 450 nm filter (OD_450nm_). Moreover, the expression of NF-*κ*B levels was also quantified by the Western blot analysis as described before [10, 11].

### 2.6. Estimation of DNA Injury by a Comet Assay (Single-Cell Gel Electrophoresis)

In separate mice (*n* = 20), the lungs collected at the end of the one-month exposure duration to either air or P-WPS or AF-WPS or SF-WPS were used to quantify the DNA injury by a comet assay (also called single-cell gel electrophoresis). The latter analysis was performed on fresh lungs immediately after their collection, as reported before [10, 15–17]. Briefly, straightway after animal sacrifice, the lungs were removed from each animal and washed in a chilled medium (RPMI 1640, 15% DMSO, 1.8% (*w*/*v*) NaCl). The lung tissues were positioned in a 1.5 ml medium and cut finely into pieces in a Petri dish. The slices were deposited and the supernatant was harvested in a 15 ml tube. The harvested cell suspension was spun at 1000 rpm for 5 min at 4°C. The supernatant was taken out and the pellets were suspended in the medium. The cell suspensions were merged with low melting point agarose solution and dispersed onto agarose-precoated microscope slides. To each group, 5 slides were prepared and left in ice-cold lysis buffer at 4°C for at least 1 h to eliminate the cell membranes. After the incubation, slides were put in a horizontal electrophoresis unit and incubated in electrophoresis buffer for 20 min for DNA unwinding and the expression of alkali labile sites. After that, electrophoresis was performed for 20 min at 25 V and 300 mA. The slides were then neutralized with Tris buffer for 5 min, washed with methanol, and stained with propidium iodide, as previously described [10, 15–17]. All the aforementioned stages were done in dark to avert supplementary DNA injury. The slides were put on a fluorescent microscope, and cell scoring was done. The measurement of length of the DNA migration (i.e., nucleus diameter and migrated DNA) was quantified by image analysis Axiovision 3.1 software (Carl Zeiss, Canada) [10, 15–17].

### 2.7. Statistical Analysis

The data were analysed with GraphPad Prism Version 7 for Windows software (GraphPad Software Inc., San Diego, CA, USA). Data were analysed for normal distribution using the Shapiro-Wilk normality test. Statistical analysis was performed by one-way analysis of variance followed by Dunnett's multiple comparison test. *P* values < 0.05 were considered significant.

## 3. Results

### 3.1. Airway Hyperresponsiveness to Methacholine

**Figure 1:**
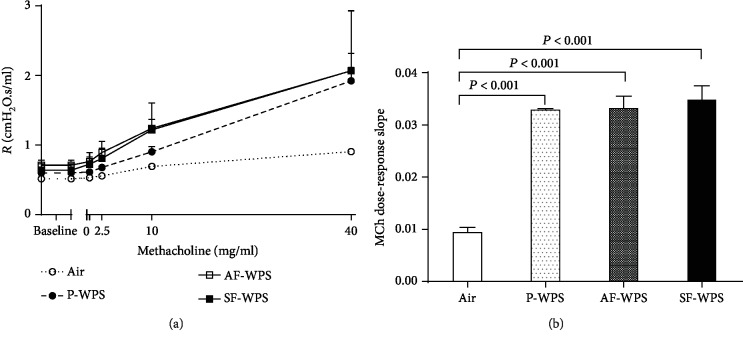
Airway hyperresponsiveness. The airway resistance (*R*), after increasing concentrations of methacholine (MCh; 0–40 mg/ml), was measured via the forced oscillation technique (flexiVent), at the end of a one-month exposure period to either air (control; *n* = 7) or plain (P-) waterpipe smoke (WPS; *n* = 6) or apple-flavoured (AF-) WPS (*n* = 5) or strawberry-flavoured (SF-) WPS (*n* = 7). Dose-response relationship of total respiratory system resistance to increasing doses of MCh (a). From the resistance MCh dose-response curve in (a), an index of airway responsiveness was calculated as the slope of the linear regression using 0–40 mg/ml concentrations (b). Data are expressed as mean ± SEM.

Figure 1 shows the assessment of airway resistance determined by the forced oscillation technique after increasing concentrations of methacholine (0-40 mg/ml) in all the groups. Airway resistance was dose-dependently and significantly elevated in mice exposed to P-WPS, AF-WPS, and SF-WPS compared with the control animals exposed to air (Figure 1(a)).

From the airway resistance methacholine dose-response curve, an index of airway hyperreactivity was calculated as the slope of the linear regression using the 0–40 mg/ml concentration (Figure 1(b)). In P-WPS (0.0331 ± 0.0003, *P* < 0.001), AF-WPS (0.0334 ± 0.0024, *P* < 0.001), and SF-WPS (0.0351 ± 0.0027, *P* < 0.001), the methacholine dose-response slope was significantly elevated compared with control animals exposed to air only (0.0096 ± 0.0010).

### 3.2. Lung Histopathology

**Figure 2:**
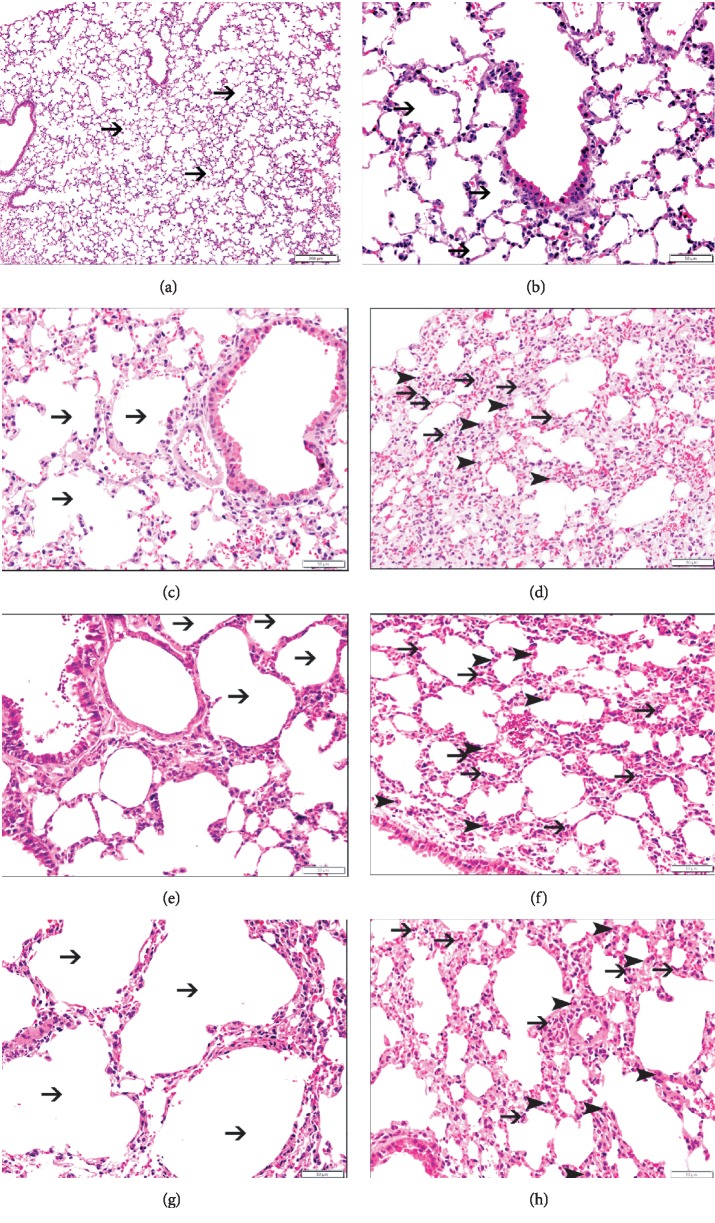
Representative light microscopy sections of lung tissues of mice, at the end of a one-month exposure period to either air (control) or plain (P-) waterpipe smoke (WPS) or apple-flavoured (AF-) WPS or strawberry-flavoured (SF-) WPS. (a, b) The air-exposed group shows normal lung tissue with uniform alveolar spaces (thin arrow). (c) The P-WPS group displays a focal area of dilated alveolar spaces (thin arrow). (d) The P-WPS group shows widening of interalveolar spaces with mixed inflammatory cells consisting of macrophages (arrowhead) and neutrophil polymorphs (thin arrow). (e) AF-WPS displays a focal area of dilated alveolar spaces (thin arrow). (f) AF-WPS shows widening of interalveolar spaces with mixed inflammatory cells consisting of macrophages (arrowhead) and neutrophil polymorphs (thin arrow). (g) SF-WPS exhibits focal area of dilated alveolar spaces (thin arrow). (h) SF-WPS shows widening of interalveolar spaces with mixed inflammatory cells consisting of macrophages (arrowhead) and neutrophil polymorphs (thin arrow).

Light microscopy examinations of H&E-stained lung sections from mice exposed to either air or P-WPS or AF-WPS or SF-WPS groups are shown in Figure 2.

**Figure 3:**
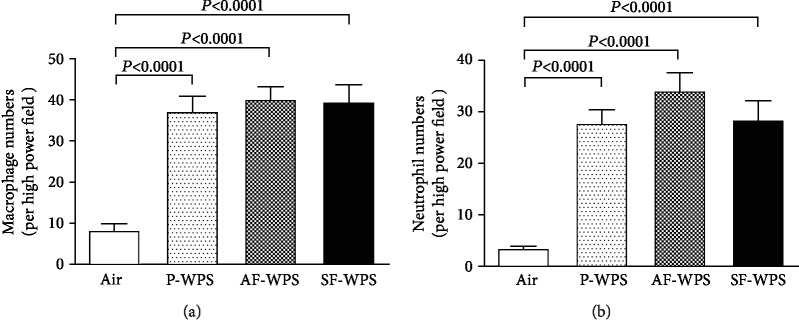
Numbers of macrophages (a) and polymorphonuclear neutrophils (b) quantified by morphometric analysis of microscopy sections of lung tissues of mice, at the end of a one-month exposure period to either air (control, *n* = 6) or plain (P-) waterpipe smoke (WPS, *n* = 6) or apple-flavoured (AF-) WPS (*n* = 6) or strawberry-flavoured (SF-) WPS (*n* = 6). Data are expressed as mean ± SEM.

The analysis of lung sections obtained from the air group revealed normal unremarkable alveolar spaces, bronchioles, and blood vessels (Figures 2(a) and 2(b)). Compared with the air-exposed group, lung sections of the P-WPS (Figures 2(c) and 2(d)), AF-WPS (Figures 2(e) and 2(f)), and SF-WPS (Figures 2(g) and 2(h)) groups showed the presence of focal areas of dilated alveolar spaces and foci of widening of interalveolar spaces with mixed inflammatory cells consisting of macrophages and neutrophil polymorphs.  Figure 3(a) shows that compared with control group (8.3 ± 1.8 cells/HPF), the number of macrophage was significantly increased in P-WPS (37.1 ± 4.0 cells/HPF, *P* < 0.0001), AF-WPS (40.1 ± 3.3 cells/HPF, *P* < 0.0001), and SF-WPS (39.5 ± 4.4 cells/HPF, *P* < 0.0001). Likewise, Figure 3(b) exemplifies that the neutrophil numbers were significantly elevated in P-WPS (27.8 ± 2.8 cells/HPF), AF-WPS (34.1 ± 3.7 cells/HPF), and SF-WPS (28.5 ± 3.8 cells/HPF) compared with the air-exposed group (3.5 ± 0.6 cells/HPF).

### 3.3. Neutrophil Elastase and MPO Activities in Lung Homogenates

**Figure 4:**
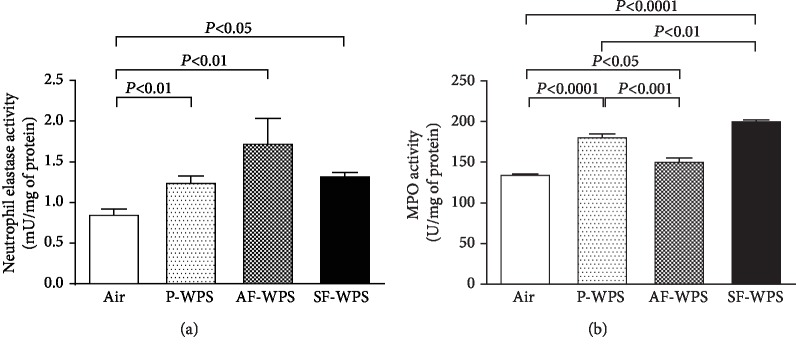
(a) Neutrophil elastase activity in lung homogenate, at the end of a one-month exposure period to either air (control, *n* = 5) or plain (P-) waterpipe smoke (WPS, *n* = 6) or apple-flavoured (AF-) WPS (*n* = 6) or strawberry-flavoured (SF-) WPS (*n* = 6). (b) Myeloperoxidase (MPO) activity in lung homogenate, at the end of a one-month exposure period to either air (control, *n* = 7) or plain (P-) waterpipe smoke (WPS, *n* = 8) or apple-flavoured (AF-) WPS (*n* = 8) or strawberry-flavoured (SF-) WPS (*n* = 8). Data are expressed as mean ± SEM.

Figure 4(a) shows that compared with the air-exposed group (0.86 ± 0.07*μ*U/mg), the exposure to either P-WPS (1.25 ± 0.09*μ*U/mg, *P* < 0.01) or AF-WPS (1.73 ± 0.31*μ*U/mg, *P* < 0.01) or SF-WPS (1.33 ± 0.04*μ*U/mg, *P* < 0.05) induced a significant increase of neutrophil elastase activity in lung homogenates.

Likewise, Figure 4(b) illustrates that the MPO activity was significantly increased in the P-WPS (181.9 ± 4.4 U/mg, *P* < 0.0001), AF-WPS (151.8 ± 4.8 U/mg, *P* < 0.05), and SF-WPS (201.5 ± 2.0 U/mg, *P* < 0.0001) groups compared with the control ones (135.7 ± 1.5 U/mg). Moreover, there were significant differences in MPO activity between P-WPS and AF-WPS (*P* < 0.001) and between P-WPS and SF-WPS (*P* < 0.01).

### 3.4. IL-6 and TNF*α* Concentrations in Lung Homogenates

**Figure 5:**
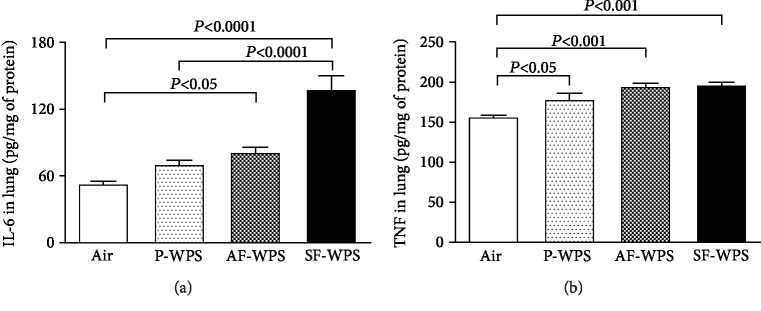
(a) Interleukin-6 (IL-6) concentrations in lung homogenate, at the end of a one-month exposure period to either air (control, *n* = 6) or plain (P-) waterpipe smoke (WPS, *n* = 6) or apple-flavoured (AF-) WPS (*n* = 6) or strawberry-flavoured (SF-) WPS (*n* = 6). (b) Tumor necrosis factor-*α* (TNF*α*) concentrations in lung homogenate, at the end of a one-month exposure period to either air (control, *n* = 7) or plain (P-) waterpipe smoke (WPS, *n* = 8) or apple-flavoured (AF-) WPS (*n* = 8) or strawberry-flavoured (SF-) WPS (*n* = 8). Data are expressed as mean ± SEM.

Compared with the control group (53.1 ± 3.3 pg/mg), the exposure to P-WPS (70.5 ± 4.5 pg/mg) did not significantly increase the concentration of IL-6 in lung homogenates (Figure 4(a)). However, the exposure to either AF-WPS (81.3 ± 5.5 pg/mg, *P* < 0.05) or SF-WPS (137.9 ± 13.5 pg/mg, *P* < 0.0001) induced a significant increase in IL-6 concentrations in lung homogenates (Figure 5(a)). Moreover, there was a significant difference in the IL-6 concentration in lung homogenates between the P-WPS and SF-WPS (*P* < 0.0001) groups.

Figure 5(b) exhibits that TNF*α* concentration in lung homogenates was significantly elevated in the P-WPS (178.6 ± 8.9 pg/mg, *P* < 0.05), AF-WPS (194.9 ± 5.1 pg/mg, *P* < 0.001), and SF-WPS (196.5 ± 4.4 pg/mg, *P* < 0.001) groups compared with the air-exposed group (156.9 ± 3.3 pg/mg).

### 3.5. Total NO and GSH Concentrations and Catalase Activity in Lung Homogenates

**Figure 6:**
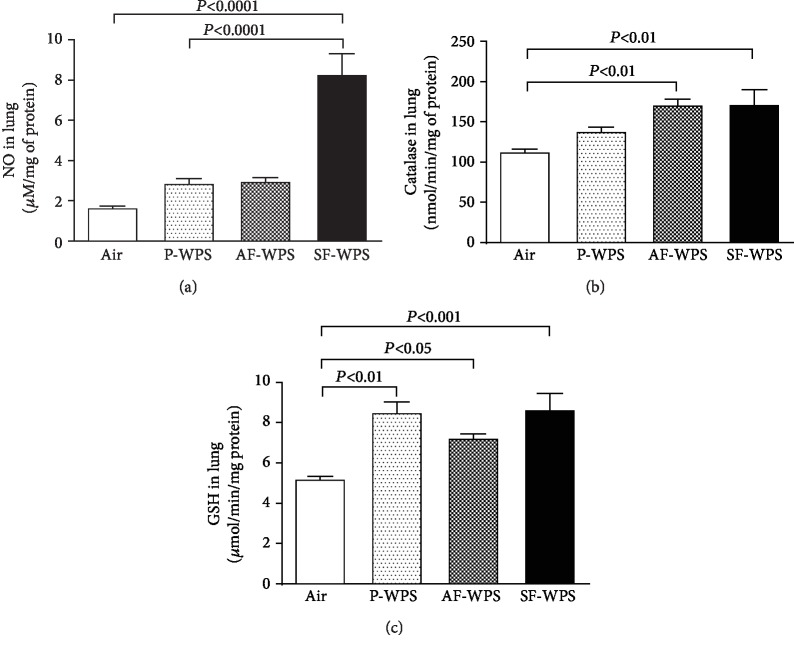
(a) Total nitric oxide (NO) activity in lung homogenate, at the end of a one-month exposure period to either air (control, *n* = 6) or plain (P-) waterpipe smoke (WPS, *n* = 5) or apple-flavoured (AF-) WPS (*n* = 5) or strawberry-flavoured (SF-) WPS (*n* = 6). (b) Catalase activity in lung homogenate, at the end of a one-month exposure period to either air (control, *n* = 5) or plain (P-) waterpipe smoke (WPS, *n* = 6) or apple-flavoured (AF-) WPS (*n* = 6) or strawberry-flavoured (SF-) WPS (*n* = 6). (b) Catalase and (c) reduced glutathione (GSH) concentrations in lung homogenate, at the end of a one-month exposure period to either air (control, *n* = 6) or plain (P-) waterpipe smoke (WPS, *n* = 6) or apple-flavoured (AF-) WPS (*n* = 6) or strawberry-flavoured (SF-) WPS (*n* = 6). Data are expressed as mean ± SEM.

Compared with the air group (1.67 ± 0.11 *μ*M/mg), the exposure to either P-WPS (2.89 ± 0.26 *μ*M/mg) or AF-WPS (2.97 ± 0.23 *μ*M/mg) induced a slight and statistically insignificant increase in total NO in lung homogenates (Figure 6(a)). Nonetheless, the exposure to SF-WPS (8.3 ± 1.0 *μ*M/mg, *P* < 0.0001) caused a significant increase in total NO concentration in lung homogenates (Figure 6(a)). Moreover, there was a significant difference in total NO concentration in lung homogenates between P-WPS and SF-WPS (*P* < 0.0001).

Compared with the control group (112.7 ± 5.0 nmol/min/mg), catalase activity was significantly increased by the exposure of mice to AF-WPS (171.0 ± 8.8 nmol/min/mg, *P* < 0.01) and SF-WPS (171.7 ± 19.6 nmol/min/mg, *P* < 0.01) but not by P-WPS (138.3 ± 6.5 nmol/min/mg) (Figure 6(b)).

However, the concentration of GSH in lung homogenates was significantly augmented in the P-WPS (8.5 ± 0.5 *μ*mol/min/mg, *P* < 0.01), AF-WPS (7.2 ± 0.2 *μ*mol/min/mg, *P* < 0.05), and SF-WPS (8.6 ± 0.8 *μ*mol/min/mg, *P* < 0.001) groups compared with the air-exposed (5.2 ± 0.1 *μ*mol/min/mg) group (Figure 6(c)).

### 3.6. DNA Damage and Cleaved Caspase-3

**Figure 7:**
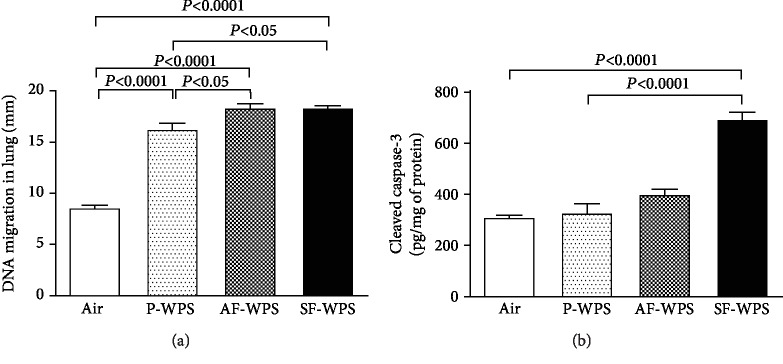
(a) DNA migration (mm) in the lung tissues evaluated by a comet assay, at the end of a one-month exposure period to either air (control, *n* = 5) or plain (P-) waterpipe smoke (WPS, *n* = 5) or apple-flavoured (AF-) WPS (*n* = 5) or strawberry-flavoured (SF-) WPS (*n* = 5). (b) Cleaved caspase-3 concentration in lung homogenate, at the end of a one-month exposure period to either air (control, *n* = 6) or plain (P-) waterpipe smoke (WPS, *n* = 6) or apple-flavoured (AF-) WPS (*n* = 6) or strawberry-flavoured (SF-) WPS (*n* = 8). Data are expressed as mean ± SEM.

As shown in Figure 7(a), compared with the air-exposed group (8.6 ± 0.3 mm), exposure to either P-WPS (16.2 ± 0.6 mm, *P* < 0.0001) or AF-WPS (18.3 ± 0.5 mm, *P* < 0.0001) or SF-WPS (18.3 ± 0.3 mm, *P* < 0.0001) induced a significant increase in DNA migration. Furthermore, DNA damage was significantly increased in AF-WPS compared with P-WPS (*P* < 0.05), and in SF-WPS compared with P-WPS (*P* < 0.05).

Figure 7(b) illustrates that compared with the control group (310.9 ± 11.7 pg/mg), only the concentration of cleaved caspase-3 in lung homogenates was significantly increased following exposure to SF-WPS (693.8 ± 31.3 pg/mg, *P* < 0.0001). Both plain (327.2 ± 40.2 pg/mg) and AF-WPS (399.7 ± 25.5 pg/mg) did not significantly increase the concentration of caspase-3. Additionally, cleaved caspase-3 concentration was significantly increased in the SF-WPS group compared with that in the P-WPS group (*P* < 0.0001).

### 3.7. NF-*κ*B in Lung Homogenate

**Figure 8:**
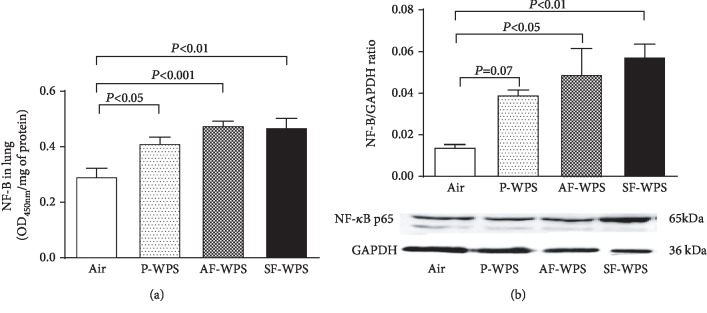
(a) Nuclear factor kappa-B (NF-*κ*B) levels in the lung tissues quantified by ELISA, at the end of a one-month exposure period to either air (control, *n* = 7) or plain (P-) waterpipe smoke (WPS, *n* = 7) or apple-flavoured (AF-) WPS (*n* = 7) or strawberry-flavoured (SF-) WPS (*n* = 6). (b) Western blot analysis and graphic representation of NF-*κ*B protein levels in the lung tissues, at the end of a one-month exposure period to either air (control, *n* = 5) or plain (P-) waterpipe smoke (WPS, *n* = 5) or apple-flavoured (AF-) WPS (*n* = 5) or strawberry-flavoured (SF-) WPS (*n* = 5). Data are expressed as mean ± SEM.

Figure 8(a) shows that exposure of mice for one month to either P-WPS (0.41 ± 0.02 OD_450nm_/mg, *P* < 0.05) or AF-WPS (0.47 ± 0.02 OD_450nm_/mg, *P* < 0.001) or SF-WPS (0.47 ± 0.03 OD_450nm_/mg, *P* < 0.01) induced a significant augmentation of the transcription factor NF-*κ*B levels in the lung compared with the air-exposed group (0.29 ± 0.03 OD_450nm_/mg).  Figure 8(b), which displays the analysis of NF-*κ*B by a Western blot, shows that compared with that of the air-exposed control group (0.014 ± 0.001), inhalation of P-WPS induced a statistically insignificant increase of NF-*κ*B expression (0.039 ± 0.002, *P* = 0.07), and a significant increase in the expression of NF-*κ*B in the AF-WPS (0.049 ± 0.012, *P* < 0.05) and SF-WPS (0.057 ± 0.006, *P* < 0.01) groups.

## 4. Discussion

The present study provided experimental evidence that exposure to P-WPS or AF-WPS or SF-WPS induces adverse alterations in lung function and morphology and causes oxidative stress and inflammation via mechanisms that include activation of NF-*κ*B. Broadly, the deleterious actions of flavoured tobacco WPS, in particular SF, were found to be significantly greater than those of unflavoured WPS.

The presence of flavourings in tobacco used in WPS is known to have boosted its popularity and attracted non-waterpipe smokers (especially the youth) to start smoking WPS believing that flavoured tobacco products are safer than other tobacco products [2]. The analysis of flavouring constituents found in waterpipe tobacco revealed the presence of seventy-nine volatile flavour compounds, eleven of which have been quantitatively analyzed, and shown to contain large quantities of the fragrance benzyl alcohol besides substantial levels of limonene, linalool, and eugenol, which are well known to be allergenic [18]. However, clinical and experimental studies comparing the toxicity of the different commercially available flavouring (or flavouring constituents) on lung physiology are scarce and much needed.

Clinical studies have demonstrated that WPS caused an augmentation of airway reactivity to mannitol and pulmonary inflammation [6]. It has also been reported that WPS inhalation in mice causes lung inflammation and increases airway hyperresponsiveness to methacholine [10, 19]. However, there is a lack of detailed information on the chemical constituents and adverse or toxic actions of flavours used in WPS on pulmonary function and morphology. Our study shows that the exposure to either P-WPS or AF-WPS or SF-WPS induced a significant and a dose-dependent increase in airway hyperresponsiveness to methacholine. Likewise, histological analysis of the lungs revealed the presence of focal areas of dilated alveolar spaces and foci of widening of interalveolar spaces with mixed inflammatory cells consisting of macrophages and neutrophil polymorphs. The latter morphological adverse changes were associated with the increase in the activities of neutrophil elastase and MPO in lung homogenates. MPO and neutrophil elastase play a key role in triggering and developing lung inflammation [20]. They are involved in neutrophil influx to the inflammatory site, where the recruited activated neutrophil polymorphs degranulate, discharging additional elastolytic proteases that induce lung tissue injury [20]. Increases in the activities of elastase, myeloperoxidase, and matrix metallopeptidase 9 have been reported, following exposure to WPS, cigarette, and e-cigarettes [8, 21, 22].

To further clarify the mechanisms by which the plain and the flavoured WPS exert their pulmonary pathophysiological effects, two markers of inflammation, viz., IL-6 and TNF*α*, and three markers of oxidative stress, viz., total NO, catalase, and GSH, were measured. The proinflammatory cytokines IL-6 and TNF*α* have been reported to increase, following exposure to cigarette smoke and WPS, and to play a significant role in the acute smoke-induced inflammatory reaction in the lung, occasioning connective tissue damage which ultimately leads to emphysema [10, 23–26]. The present data show that the exposure to P-WPS did not increase the concentration of IL-6 in lung homogenates. However, the exposure to either AF-WPS or SF-WPS induced a significant increase in IL-6 concentrations. On the other hand, TNF*α* concentration in lung homogenates was significantly elevated in all the plain and the flavoured WPS. It is well recognized that exposure to cigarette smoking or WPS induces oxidative stress that results from a disproportion between prooxidant and antioxidant owing to elevation of endogenous reactive oxygen species released by neutrophils, macrophages, and pulmonary cells such as alveolar epithelial and endothelial cells [10, 19]. It is also well established that cells can be protected against oxidative stress by enzymatic and nonenzymatic antioxidant systems that can augment the endogenous antioxidant system and reduce oxidative stress [27]. Along with the increase of inflammation, it was observed that the increase of total NO in lung homogenate was only seen in mice exposed to SF-WPS and the augmentation of the activity of antioxidant catalase was recorded in mice exposed to either AF-WPS or SF-WPS. However, the GSH concentrations were increased in P-WPS, AF-WPS, and SF-WPS. The reason why P-WPS did not cause an increase in the concentration of IL-6, total NO, or catalase cannot be readily explained, but this finding clearly suggests that the flavouring added to the tobacco used in WPS increases the inflammatory and oxidative impact of WPS. Further studies are required to clarify this point. In line with our findings, it has been shown that vapours produced by e-cigarettes and e-juices with flavourings induce toxicity, oxidative stress, and inflammatory response in lung epithelial cells and in the mouse lung [28]. Moreover, it has been recently demonstrated that exposure of human monocytic cell lines to e-cigarette flavouring chemicals induced an increase of proinflammatory cytokine IL-8 and reactive oxygen species and that some flavours and their main flavouring chemicals induced more cytotoxicity than others [29]. In fact, it was found that cinnamaldehyde, vanillin, and pentanedione were the most toxic flavouring chemicals on monocytes [29].

Augmented oxidative stress has been directly related to oxidation of protein DNA and lipids, which could induce direct lung injury [27]. It has been shown that exposure to diesel exhaust particles or WPS induces DNA oxidation injury resulting from oxidative stress and inflammation [8, 30]. The present data show that the exposure to either P-WPS or AF-WPS or SF-WPS caused lung DNA damage. It is well known that oxidative DNA damage caused by reactive oxygen species can eventually result in cellular dysfunction and apoptosis [31]. Apoptotic mediators such as the caspases are key mediators of apoptosis, and caspase-3 is a commonly actuated death protease, catalysing the cleavage of various key cellular proteins [31]. The present data show that compared with the control group, there is a statistically insignificant increase of cleaved caspase-3 concentration in the P-WPS and AF-WPS groups and that the levels of cleaved caspase-3 observed in SF-WPS were higher than those seen in the control and P-WPS groups. The latter result could be explained by the overall higher impact of SF-WPS on oxidative stress (increase of NO, catalase, and GSH) compared with either AF-WPS (increase of catalase and GSH) or P-WPS (increase of GSH). The occurrence of apoptosis along with DNA damage and oxidative stress has been reported previously in the lung of mice exposed to diesel exhaust particles and in the heart of mice exposed by inhalation to WPS [30, 32].

NF-*κ*B is located in the cytosol in an inactive form attached to its inhibitory protein I*κ*B. Various stimuli, comprising cytokines and oxidants, can activate NF-*κ*B, ensuing in ubiquitination cleaving of I*κ*B from NF-*κ*B and the destruction of I*κ*B in the proteasome [33]. The activation of the NF-*κ*B signalling pathway induces upregulations of proinflammatory cytokines including TNF*α*, IL-6, and IL-1*β* and oxidative stress, and pharmacological approaches are being developed to block this transcription factor to prevent inflammation and oxidative stress-associated pulmonary diseases [34]. The current data show that together with the occurrence of inflammation, oxidative stress, and DNA damage, the exposure to either P-WPS or AF-WPS or SF-WPS induces a significant increase in the levels of NF-*κ*B. This finding is in agreement with recent studies which showed the increase of expression of NF-*κ*B in the lung of mice exposed to diesel exhaust particles or WPS [10, 30]. It has also been demonstrated that exercise training and nootkatone, a constituent of grapefruit that has antioxidant and anti-inflammatory effects, significantly alleviate the activation of NF-*κ*B [12, 24].

The present study has limitations. The levels of NF-*κ*B were quantified from the whole lung homogenate, and hence it is not clear in which specific cell types NF-*κ*B was increased. Also, there was no comparison between the nuclear and cytoplasmic levels of NF-*κ*B. Moreover, in this study, the assessment of markers of inflammation was confined to IL-6 and TNF*α* and those of oxidative stress were limited total NO, catalase, and GSH. Further studies are warranted to assess more markers of inflammation and oxidative stress and to investigate the chronic impact of flavouring on the lung function and morphology.

Taken together, the present data demonstrate that that exposure to unflavoured WPS or AF-WPS or SF-WPS induces alteration in lung function and morphology and causes oxidative stress and inflammation via mechanisms that include activation of NF-*κ*B. Overall, the toxicity of flavoured tobacco WPS, in particular SF-WPS, was found to be greater than that of unflavoured WPS. Additional studies are required to evaluate further the impact of flavouring chemicals used in WPS on lung physiology and to identify the specific agents responsible for the deleterious pulmonary damage. The current experimental findings provide biological plausibility for the harmfulness of flavoured tobacco used in WPS and support calls for interventions to counteract the increase of attractiveness and use of WPS, particularly among young people.

## Data Availability

The data that support the findings of this study are available from the corresponding author, Abderrahim Nemmar, upon reasonable request.
